# Qualification of Staff, Organization of Services, and Management of Pregnant Women in Rural Settings: The Case of Diema and Kayes Districts (Mali)

**DOI:** 10.5402/2012/649412

**Published:** 2012-05-03

**Authors:** Maman Dogba, Pierre Fournier, Safoura Berthe-Cisse

**Affiliations:** ^1^Department of Public Health, Faculty of Medicine, University of Montreal, 1420 Mont-Royal Boulevard, Montreal, QC, Canada H2V 4P3; ^2^International Health Unit, Research Center of the Centre Hospitalier de l'Université de Montréal (CRCHUM), 3875 Saint-Urbain Street, Montreal, QC, Canada H2W 1V13; ^3^Direction Régionale de la Santé, P.O. Box 231, Kayes, Mali

## Abstract

In Mali, a poor sub-Saharan country, maternity referral systems were implemented to combat the still-high rates of maternal mortality. This qualitative study was aimed at understanding the relationships between the qualification of staff in community health centres, the organization of services, and the management of pregnant women in the maternity referral system in Kayes, a rural region of Mali. Physicians who managed CHCs actively or passively modified work organization, the level of technology, their obstetric skills, and staffing. They also created a competitive environment and developed relationships of trust with patients and with the district health centre. These findings are helpful in orienting decision-making for better personnel management.

## 1. Introduction

The human resources crisis created by personnel shortages is currently the greatest impediment to generalizing proven strategies for improving maternal and perinatal mortality [1–4]. In Mali, referral-evacuation systems (RESs) are one strategy in the fight against maternal and perinatal mortality, where the implementation has had to cope with shortages of staff, particularly of midwives in rural settings. An RES looks after the upgrading of emergency obstetric services, the financial coverage of patients by community cost-sharing schemes, and the transfer of patients, as needed, to better equipped facilities [5]. It closely links peripheral healthcare structures (community health centres (CHCs))), district health centres (DHCs), and regional hospitals. It is organized around the district, the basic functional unit of Mali's healthcare system [6]. Its interventions are concentrated on the intrapartum period, during which most maternal deaths occur [7]. It includes a functional referral and allows for simultaneous action on the demand and supply sides. Indeed, the RES ensures rapid management of obstetric complications in Mali's context, which is characterized by low rates of institutional delivery, especially in rural settings. In 2006, the rates of facility-based deliveries were 45% in Mali, 34% in Kayes, and 90.3% in Bamako, the national capital [8]. 

Mali, a low-income West African country, has eight administrative regions. RESs have been implemented in all of them, with considerable variation in the levels of inter- and intraregional functionality. In Kayes region, where this study was conducted, the seven districts' RESs were set up between 2002 and 2005. They are supposed to facilitate normal deliveries in the CHCs and the referral of complicated cases to the DHCs. For early screening and referral of obstetric complications, whose occurrence is most often unpredictable, all deliveries should be carefully monitored by staff qualified for deliveries—essentially either midwives or other comparably skilled professionals [9, 10]. Thus, in Kayes region, counting one midwife per CHC, 223 midwives would be required to implement the RESs, in addition to those at the DHCs and the regional hospital. However, in 2005, there were only seven midwives at the CHC level, and, in 2006 and 2009, only eleven. In the face of these shortages of qualified personnel, two strategies were implemented. One that took immediate effect was to extend obstetric services coverage in CHCs by using matrons. They are much less qualified than midwives but are more easily trained, recruited and, especially, retained in rural areas because they come most often from the local communities that train and employ them. Midwives complete three years of study after their baccalaureate degree, whereas matrons undergo a few months of internship and some theoretical training in a maternity unit. The content of the matrons' training varies depending on the maternity unit they attend. Moreover, no particular level of schooling is required for this training, although most matrons have at least completed primary school. In 2005, Kayes region had 196 matrons in CHCs and 222 in 2006. Another strategy was to train obstetric nurses, who are less qualified than midwives but more than matrons. They are considered qualified to do deliveries [12, 13]. They receive three years of theoretical and practical training in competencies similar to midwifery skills, after a basic teaching diploma (six years of primary and four years of secondary schooling). In the CHCs of Kayes region, there were eight in 2005 and 14 in 2006. Despite these compromises in staff qualification at the peripheral levels, the implementation of RESs doubled the number of women who had access to obstetric care and reduced by half their risk of dying from obstetric complications. These results were made possible through the combined action of all components of the RES [5].

In addition to the shortages of midwives, the numbers and qualifications of staff in the CHCs' healthcare teams vary considerably. In Kayes region, as everywhere in Mali, CHC management falls under the responsibility of local communities and is conducted through community health associations (ASACO) that can take the initiative in staff recruitment and remuneration [6, 14]. Kayes region is the largest source of Malian emigrants to France [15], and the ASACOs enjoy the financial support of Malian citizens from Kayes who are living abroad. Healthcare projects are sometimes funded as much as 80% by emigrants from the Kayes region, which represents 30% of the combined total contributions to the region's development [16]. Thus, while some CHCs might have only three professionals—a nurse, a matron and a pharmacy manager—others might have as many as eight professionals. In addition, around 10% of the CHCs in the Kayes region have physicians. In CHCs where there is a physician, that person is automatically the centre's manager: the physician-in-charge (*médecin chef de poste*, or MCP). Otherwise, it is the most qualified staff, often the nurse, who is the manager: the nurse-in-charge (*infirmier chef de poste*, or ICP).

A quantitative evaluative study looked at the relationships between professional teams and care outcomes. It showed that the joint mother-newborn survival is significantly influenced in the Kayes maternal referral system by combined effects of the skill configuration of CHC personnel and distance traveled. Thus, women referred from a CHC where there was a physician were six times more likely to survive from an obstetric complication than were those transferred from a CHC without a physician, based on comparable morbidity and controlling for distance travelled and other cofactors [17]. Pregnancies complications were not managed at the CHC, but, at the DHC, the protective role of physicians' presence in CHCs was therefore attributed to a better management of complicated cases before their evacuation [18, 19], or to earlier screening of women potentially at risk of obstetric complications, or to the beneficial effects of ambulance transport. Despite those hypotheses, a more rigorous understanding is needed of the mechanisms that link the management of pregnancies, the qualification of staff, and the organization of services at the first level of care provision, especially at a time when shortages of qualified personnel lead to the adoption of task-delegation strategies [20–23].

## 2. Methodology

We conducted a multiple-case study in the districts of Kayes and Diema. Using purposive and stratified sampling, we selected, in two stages, 25 CHCs from the districts' total of 67 CHCs as units of analysis. First, we selected 13 CHCs headed by physicians (MCPs), taking into account the number of healthcare personnel and the distance between each CHC and the DHC, to maximize variability in the care environment. Then, we selected 12 other CHCs headed by nurses (ICPs); these were comparable to the MCP-managed CHCs in every respect except for the qualification of the manager in charge.

We carried out semistructured interviews with the personnel involved in maternal care: matrons, nurses, obstetric nurses, midwives, and physicians. In each CHC we visited, participants were invited to freely elaborate about the organization of maternal services in their centre and about what they did to improve the outcome of care for the women. This enabled us to develop a picture of the centre's functioning and to validate it with the staff. Through nonparticipant observations, we were able to observe professional interactions in the teams and to verify whether the organizational modalities corresponded to what was stated in the interviews. The interviews were carried out by the first author and a sociologist from the region. On average, the interviews lasted 60 minutes (between 45 and 90 minutes). We interviewed ICPs (*n* = 10) (two ICPs were absent from their posts and one interview with an ICP was discarded because of technical problems with the recording), nurses (*n* = 3), matrons (*n* = 23), midwives (*n* = 5), obstetric nurses (*n* = 4), and MCPs (*n* = 11) (one MCP had just been named to his post and was not interviewed). In all, we conducted 56 interviews and observed for 10 days.  Table 1 shows the characteristics of respondents.

The interviews were recorded with the respondents' consent and then transcribed. The coding and analysis of the transcribed material were done using QSR International's NVivo 8 software. We developed the coding plan from a list of codes inspired by the literature on human resources in healthcare services and on quality of services [24]. The list was combined with an open coding to allow the emergence of new themes. Units of meaning were identified according to the predefined or emergent themes. We then compared the units of meaning between professional categories and within each category. Comparisons between MCPs and ICPs, reported in this study, allowed us to identify points in common as well as differences. 

### 2.1. Ethical Considerations

This project received ethical approval from the Research Centre of the University of Montreal Hospital Centre, from Mali's National Department of Health, and from the Kayes Regional Department of Health. In accordance with local practices, verbal informed consent was obtained from the ASACO managers and health personnel for the interviews as well as for their recording. To maintain respondents' anonymity, extracts from the interviews are reported using identification numbers.

## 3. Results

*Variations in the care environment* include how work is organized, the level of technology, the skill and numbers of staff in the CHC, and the creation of a competitive environment.

### 3.1. Variations in Work Organization

In the 13 CHCs with ICPs, the distribution of tasks for the clinical management of pregnant women followed a homogeneous model, while those with MCPs showed a variety of profiles.

The ICPs looked after general consultations, oversaw the proper functioning of the CHC, and delegated responsibility for maternity activities to the matrons. The matrons organized prenatal consultations and did the deliveries; they sought technical advice from the ICPs for complicated cases. The ICPs also coordinated patient transfers to DHCs. In these CHCs, the ICP rarely took the initiative to monitor simple deliveries. The following excerpt illustrates this work organization, which was nearly uniform in all CHCs headed by nurses:

“When there is no complication, the matron is in charge, but under my supervision.” (ICP_10).

In some CHCs managed by ICPs, this division of tasks, which was sometimes associated with a physical separation between the maternity unit and other buildings, led to the perception that the CHC was made up of two distinct entities:

“the first matron is our boss… and the ICP is the boss of everyone… She (the matron) is the person that the women listen to and are influenced by the most.” (Matron_44).

Analysis of the organization of work in CHCs with MCPs shows three models of service organization.

The first model is comparable to CHCs managed by ICPs and was found in five CHCs with MCPs. Matrons managed deliveries and only called on the MCP in serious cases. Despite only being directly involved in a selective and limited way with the serious cases, the MCPs did systematic telephone followup of patients with the matrons.

In the second model, seen in three CHCs, the MCP's involvement with pregnant women was more frequent and involved both simple and complicated cases. This model was characterized by two complementary measures: the systematic examination of women by at least two members of the personnel, and the organization of weekly staff meetings and presentations where the week's difficult cases were discussed. Two of these MCPs said they implemented these measures to improve the knowledge of their team members and to provide patients with appropriate management of complicated cases.

“…we divide the work; the nurse looks after the prenatal consultation, and in real time, if a woman arrives, the matron can look after her. (Even) if she is very busy, the nurse sees the woman, *systematically*, even if she has to let the matron continue.” (MCP_35).

The third model of work distribution seen in three MCPs was characterized by their very strong involvement in the management of pregnant women. They conducted the first examinations of many women and sometimes did deliveries, even for simple cases.

“Yes, I'm there for all the deliveries. Often at night I do not wake the matron, I do the deliveries myself. I am the “gynecologist.” (MCP_48).

### 3.2. Variations in Levels of Technology

In a context in which CHCs are managed autonomously by the ASACOs, the possibility of having MCPs with obstetric skills was accompanied by the purchase of ultrasound equipment in three of the CHCs that had physicians. In addition, with the support of some Kayes emigrants, two CHCs were fitted with operating suites and were in negotiations with the Regional Department of Health to obtain authorization for interventions. Expertise in the use of ultrasound was one of the recruitment criteria for staff in these facilities and was helpful for the early diagnosis of certain pathologies, as this physician explained.

“When a patient arrives who has had no prenatal visits, I quickly do an ultrasound, and this lets me know if it is a case of twins, or of placenta praevia, and we can make decisions quickly.” (MCP_18).

While no ICPs envisioned raising the level of technology in their centres—since according to them, the complicated cases should be referred to the DHCs—the MCPs were preparing development plans for their CHCs. In these development plans, the MCPs envisioned raising the levels of emergency obstetric care (EmOC) competency in their teams, setting up a functional laboratory in addition to ultrasound, installing internet connections, promoting the use of solar energy, and, in the longer term, creating functional operating suites.

“I would like to see it transformed into a referral health centre (DHC) someday, since, as you can see for yourself, it's far to refer a patient; God knows what could happen. This is why I am doubling my efforts to evacuate less. We already have ultrasound; I'm looking into how we can also set up a lab so I can do the initial analyses here.” (MCP_48).

### 3.3. Variations in the Skills and Numbers of Staff

Six MCPs had a particular interest in obstetrics which led them to acquire skills in emergency obstetric and neonatal care (EmONC). Three of them had done a thesis in a gynaecology-obstetric service, and the other three had undergone supplementary training in EmONC or in obstetric ultrasound, most often at their own expense. All of them were more deeply involved (model 2 or 3) in the management of pregnant women.

“I got my training over the Internet. When I go to France, I use my vacations to do applied training sessions with my colleagues, but it's really a personal choice.” (MCP_23).

Aside from training offered by the Regional Department of Health to upgrade their EmOC skills, the ICPs were unable to obtain any other supplementary training in obstetrics. Either they did not satisfy the conditions required for acceptance into these training programs, or they were unwilling to pay for the training themselves. None of the ICPs we interviewed had recently been able to update their EmOC skills. These training sessions competed with several others; also, they were held in the regional capital, such that the ICPs would have to travel. Staff who went for training were expected to brief the rest of the personnel. So the ICPs, knowing that the matrons would brief them and the other staff on the maternal care training they received, and wishing to limit their own absences from their posts, preferred instead to attend training sessions on HIV-AIDS and on policies, standards, and procedures.

In addition to the acquisition of additional skills, the presence of MCPs in CHCs was associated with staff recruitment. Nursing students preferred doing training internships in CHCs where there was an MCP in order to learn more. In addition, with the support of the MCP, these trainees were able to negotiate a contract as volunteers at the end of their studies. This ability to attract personnel changed the staffing levels in the CHCs, the workloads, and the combination of skills available for maternal services.

We encountered only one midwife in an ICP-managed CHC. All the other midwives and obstetric nurses were in CHCs managed by MCPs. However, they asserted that they preferred these centres, not because of the presence of a physician, but because they were high-volume centres, so they would not risk losing their skills. Some midwives regretted never having had the opportunity in the CHCs to practise even the simplest emergency procedures. The presence of midwives and obstetric nurses in the team improved the combination of skills available for maternal care; however, having female staff whose families did not live in the CHC's village complicated human resources management because of absences due to family reasons, as illustrated by the following excerpt.

“But the only problem is the instability of the ON (obstetric nurse), who spends one week here and three weeks in Bamako” (Nurse_36 in an MCP-managed CHC).

### 3.4. Creation of a Competitive Environment

The presence of physicians in the CHCs of Kayes Region created a competitive services environment that could, directly or indirectly, provide incentives for professionals' performance.

“…since, as you know, in this district there are a lot of physicians, there's competition; if you're not competent, the villagers will go elsewhere for their care.” (MCP_18).

The MCPs reported benchmarking practices. To improve their performance, they compared themselves against best practices in healthcare in the region. On their own initiative, some arranged informally to take introductory courses on ultrasound from other colleagues in the region. Some collective initiatives were also mentioned.

“We even went to K… with the members of the ASACO, to exchange ideas and experiences in the context of advancing the CHC. Because K… is a CHC that does a lot. So, within the framework of exchanging ideas, we went there.” (MCP_57).

*To develop the relational and interpersonal aspects of care*, the MCPs paid particular attention to relations with the professionals of the DHCs and with the patients.

### 3.5. Relations with the Professionals of the DHCs

The ICPs see interactions between the CHCs and the DHCs as administrative relations that should be maintained but should not influence the management of referred patients. In fact, the DHC was “the trustee organization” (ICP_1) and “the decider” (ICP_24); “it ensures the proper functioning of the CHCs through the supply of vaccines and other materials and it receives quarterly reports from the health information system.” (ICP_1)

Only one MCP stated that relations between the two levels of care had no influence at all on the management of pregnant women. All the other MCPs considered it crucial to maintain good relations with the DHC to ensure better management of referred patients. Some MCPs nurtured relationships with the higher level of care that were sometimes already preferential.

“… our connections with the referral centre (DHC) are excellent because, as I said, these are colleagues, friends; they're civil servants like me, and we *have to work together to get results*… with regard to the women who are evacuated, generally they give us feedback…” (MCP_35).

“…when I evacuate someone to …, where I have good connections, and I'm in contact with everyone, so then I just make a phone call to get whoever is on call to refer my patient, who is quickly taken on. I refer my people and they take care of them. I think it's very important to be on good terms with them.” (MCP_18).

The advantages to patients of privileged relations with the higher level of care were confirmed by a midwife who had previously worked in a DHC.

“…it will be better if women go first to the CHC. When you arrive with a referral letter, they take care of you faster, they do not just leave you hanging.” (Midwife_32).

### 3.6. Relations with Patients

According to the MCPs, establishing a relationship of trust with the population made it possible to mobilize all the resources of the village and facilitated women's acceptance of medical recommendations, such as transfers to DHCs. Unlike the ICPs, who did not mention the importance of this relational aspect, the MCPs reported that they made an effort to gain people's confidence.

“If the population is not informed and aware, we won't have good results…I've gained people's confidence, we understand each other, and when I say something, they do it…” (MCP_35).

In summary, the variations identified in the MCPs' care environment were sometimes deliberate and actively induced—changes in the way work was organized, the level of technology, and the qualifications of the personnel—and sometimes they arose passively and unintentionally. The results of this analysis are summarized in Table 2.

## 4. Discussion

Analysis of the functioning of CHCs shows that MCPs, depending on their interests in obstetrics, had varying levels of involvement in the management of deliveries. On the other hand, in CHCs with ICPs, the organization of work was uniform and conformed to the official model provided in the RES. More frequent, direct involvement of a physician in the management of pregnant women improved the combination of skills applied in maternal services. It is true that people perform better in contexts that correspond to their personal preferences [25], and, as this study shows, the MCPs with the greatest involvement were those who had supplementary training in obstetrics. Still, an interest in obstetrics alone does not explain the development of diverse models. In fact, even when they organized the work like the ICPs did, leaving matrons in charge of deliveries, the MCPs put in place systems such as systematic phone call followup and management of patients by several professionals, to ensure complicated cases were properly managed. In this way, they were not restricted to the traditional care model of the RES used by nurses.

Also, in the present study, the ICPs spoke only about the administrative aspects of their relations with the DHC, while MCPs nurtured their relations with those working at the higher levels with whom they established personal contacts to accelerate the handover of their patients. A quick management of obstetric complications is a factor in improving the quality of obstetric care [26, 27]; nevertheless, further studies are needed to document better the consequences for patient care of the relations between the two levels of care. The MCPs also developed relationships of trust with patients, while the ICPs did not mention this. Yet, in the case of a referral system, social interactions with patients are recognized as being beneficial to patients [28]. Although giving preferential treatment to patients referred by a physician might be criticized from an ethical standpoint, in the context of a network where final outcomes depend on a succession of prior actions, an interdependent team collaboration with information exchange between the different levels is more effective than working independently, and even more effective than consultation referral [29].

In addition to going beyond the traditional model of care and developing the relational and interpersonal aspects of care, the presence of MCPs can raise the level of technology in CHCs and create a competitive environment that helps to improve performance. In a context of staff shortages where the general trend is to use less-qualified staff, the tasks, roles and responsibilities of qualified staff that are usually delegated are technical ones, such as anesthesia [21], cervical smears [30], first-trimester abortions [22], or surgical interventions [20]. However, these acts are usually carried out in the district level centers. At the peripheral level, often the responsibilities of the healthcare personnel, beyond technical interventions, include relational activities such as accompaniment and social support [31]. It would seem appropriate, therefore, to wonder whether the tasks to be delegated, especially in the health centres and in a referral system, should not also include relational and interpersonal skills. Moreover, unintentional mechanisms that improve performance, such as the capacity to attract personnel and especially to create a competitive environment [32], should be identified and maintained.

The presence of MCPs with an interest in obstetrics may improve the quality of care, but it does not resolve the problem of shortages of staff qualified to assist during women's deliveries. In fact, as this study shows, the difficulties of retaining midwives and obstetric nurses in rural areas continue, while filling these positions would have a long-term positive impact on maternal care [11, 33, 34]. Also, the environmental variations produced by physician managers veer away from the logic underlying the RES. Initially, only simple deliveries were supposed to be handled in the CHCs. However, the present study shows that, with management autonomy and support from expatriates, some CHCs have acquired increasingly qualified staff and sophisticated equipment, including operating wards. With no governmental regulation of what acts are to be done in CHCs, MCPs with obstetric competencies could take over the management of complicated cases at the most peripheral levels. However, this displacement would not be cost-effective and the number of interventions would most certainly be too small to maintain skills.

Through the study, we were able to explore relations between the qualifications of the staff heading up the CHCs, the organization of care, and the management of pregnant and child birthing women's care in rural settings. While its generalizability to other contexts is limited, this study provides some understanding, in the specific context of Kayes region, of the complexity and diversity of care organization in CHCs.

## 5. Conclusion

Kayes region (districts of Kayes and Diema) is a specific context in which innovations in service provision can be studied. Significant levels of emigration and the flow of resources back to the emigrants' communities of origin have allowed these communities to acquire a service offer that exceeds national standards [15]. A significant proportion of first-line health centres in rural settings have a physician [14]. This profoundly changes the organization of care, because physicians bring a development model to their CHCs that is centred on clinical practices and closely linked to their personal and professional development. When nurses are in charge of first-line health centres in rural areas, their approach is more administrative; they tend to adopt a more hierarchical than clinical perspective in their relations with the higher district level and a work organization model in which they delegate obstetric responsibilities to matrons. In the referral-evacuation system of Kayes region, the presence of physician managers in the CHCs created more opportunities to improve patient care outcomes. An analysis of the CHCs' models of care organization reveals organizational, relational, and interpersonal mechanisms that can improve their performance. Still, this should be an ad hoc and temporary strategy, as the problem of shortages of staff qualified for delivery—particularly midwives and obstetric nurses—continues, and there is a real risk that the referral system's operating principles would be modified in this context of autonomous management of CHCs.

## Figures and Tables

**Table 1:** Characteristics of the CHCs and the respondents.

	Kayes district	Diema district	Total	Grand total
Distribution of CHCs by manager				

CHC with MCP	11	2	13	25 CHCs
CHC with ICP	8	4	12	

Distribution of CHCs by distance between CHC and DHC				

50 km or less	7	5	12	
More than 50 km	11	2	13	

Distribution of CHCs by staff levels				
3 staff or less	8	4	12	25 CHCs
More than 3 staff	11	2	13	

Distribution of respondents by staff category				

Physicians	9	2	11	56
Midwives	5	0	5	respondents
Nurse managers	7	3	10	
Other nurses	3	0	2	
Matrons	19	4	23	
Obstetric nurses	4	0	4	

**Table 2:** Summary of variations in the environment and in work organization under MCPs.

	Technical and organization aspects				Relational and interpersonal aspects		
	Work organization	Level of technology	Staff skills	Staffing levels	Competitive environment	Relations with the DHC	Relations with patients

Intentional variations	Closer involvement in patient management	Plans to raise the level of technology	Supplementary training in obstetrics	Support for trainees	Individual or collective benchmarking	Maintained	Acquisition of their population's confidence

Unintentional variations		Equipment (operating suites, ultrasound) provided by the ASACOs with expatriate support		Attractive to trainees Midwives and obstetric nurses in centres with MCPs	Competition that stimulates performance		Patients' preference for more qualified staff
